# Strength of tremor patches along deep transition zone of a megathrust

**DOI:** 10.1038/s41598-018-22048-8

**Published:** 2018-02-26

**Authors:** Masayuki Kano, Aitaro Kato, Ryosuke Ando, Kazushige Obara

**Affiliations:** 1 Earthquake Research Institute, The University of Tokyo, Tokyo, Japan; 2 Department of Earth and Planetary Science, The University of Tokyo, Tokyo, Japan; 3 Present Address: Department of Geophysics, Graduate School of Science, Tohoku University, Sendai, Japan

## Abstract

Deep low frequency tremors are indicators of slow slip transients in the brittle-ductile transition zone along subducting plates. Investigation of comprehensive tremor activities is therefore an important issue for understanding the seismic/aseismic characteristics in transition zones. Here, we focus on the radiated energy from tremors to reveal the along-strike heterogeneity in the strength of tremor patches. Based on a tremor catalog that more accurately evaluates radiated energy, we examine the spatio-temporal activity of tremors accompanied by slow slip events (SSEs) in western Shikoku, southwestern Japan. The new finding of this study is that the energy radiated from tremors is positively correlated with the speed of tremor migration front and the slip rate along the plate interface during a SSE. This can be qualitatively explained by a stress diffusion model, which consists of along-strike heterogeneities in the effective strength of tremor patches embedded in a ductile shear zone. This effective strength heterogeneity is supported by a lateral variation in the stress drop of a SSE; it is consistent with the fluid pressure distribution along the plate boundary fault and the tidal sensitivity of tremors. Accurate evaluation of tremor activities, especially the radiated energy, can be used to infer the spatial distribution of the strength of tremor patches in transition zones worldwide.

## Introduction

Since the discovery of deep low frequency tremors along the Nankai subduction zone in western Shikoku, southwestern Japan^1^, tremor activities have been widely investigated in many subduction zones around the world^2–4^. Tremors are considered to be the seismic signal of slow earthquakes, and are often accompanied by geodetically detected slow slip events (SSEs) named as episodic tremor and slip (ETS)^5,6^. These phenomena occur in transition zones at the downdip portion of the locked seismogenic zone and thus their activities may play an important role in the occurrence of megathrust earthquakes.

Radiated energy of tremors is a fundamental parameter to understand the seismic/aseismic characteristics in transition zones and comprehensive tremor activities such as the migration speed of tremors, their relation to SSEs during the ETS episodes and the strength of tremor patches. Here, we term the strength as the total stress that the whole area of a tremor source can support. Several previous studies have evaluated tremor energy^7–9^ and discussed the heterogeneous distribution of radiated energy along the plate boundary fault^8,9^. This regional difference might reflect the heterogeneity in the strength of tremor patches; however, the estimation of radiated energy was not complete because some tremor sequences, especially high-amplitude sequences, escaped detection due to their complex waveforms radiated from almost simultaneous occurrence of intensive tremor activity. Therefore, utilizing a newly constructed tremor catalog^10^ that more accurately evaluates radiated energy, we investigate the spatio-temporal evolution of radiated energy from active tremors during major ETS episodes.

**Figure 1:**
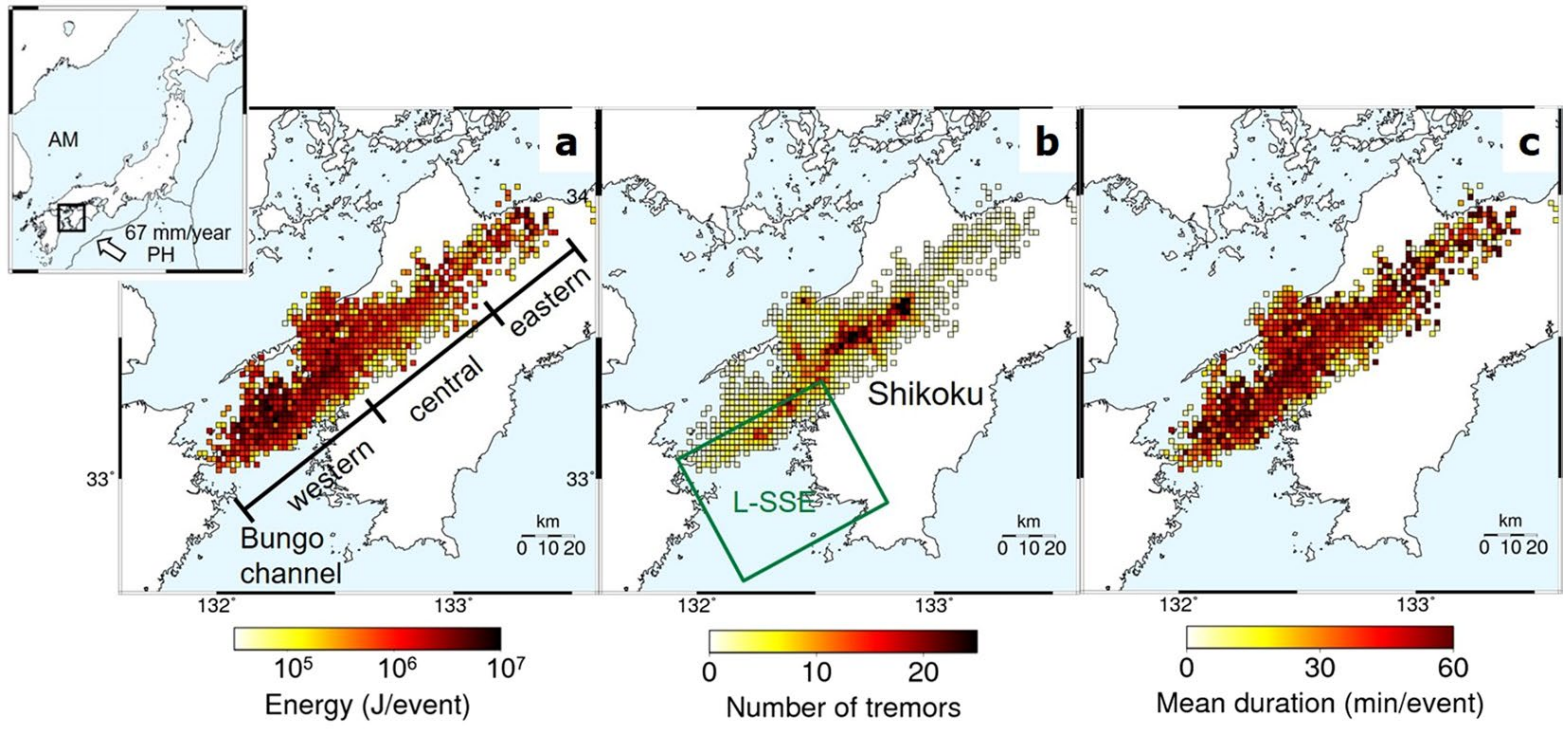
Spatial characteristics of tremor activities. Spatial distribution of (**a**), energy, (**b**) the number of tremors, and (**c**), mean duration of each tremor sequence, based on the tremor catalog^10^ during the ETS episodes from April 2004 to March 2015 in western Shikoku (the black rectangle in the upper-left inset). The study area is divided into three sub-areas. The green rectangle is the main slip region of long-term SSEs in the Bungo channel in 2010^31^. The white arrow in the inset indicates the plate motion of the subducting Philippine Sea (PH) plate relative to the Amurian (AM) plate. Maps are created using the GMT (Generic Mapping Tools, http://gmt.soest.hawaii.edu/) software package^32^.

This study focuses on western Shikoku (Fig. 1), southwestern Japan, an area of intensive tremors. Based on the new tremor catalog, we first briefly summarize the characteristics of tremor activities in this region. The detailed analysis of the spatio-temporal evolution of tremor activities clarifies that the radiated energy of tremor events is positively correlated with the migration speed during ETS episodes. Next, we show that this positive correlation can be qualitatively explained by a stress diffusion model^9^ consisting of tremor patches with a lateral heterogeneity in strength and a ductile background region. Furthermore, the along-strike contrast in strength is consistent with fluid distribution in the overriding continental plate estimated by ref.^11^, suggesting that the strength of tremor patches is controlled by the fluid pressure along the subducting plate interface.

## Spatial characteristics of tremor activities

Lateral variations of physical properties along the plate interface can be identified beneath western Shikoku from the spatial distributions of mean energy, the number of tremors, and the mean tremor duration recorded during 28 ETS episodes from April 2004 to March 2015 (Fig. 1). In the western part, which is located adjacent to the region of long-term SSEs (L-SSEs) in the Bungo channel, the radiated energy per tremor event is higher compared to other regions despite the small number of tremors. The energy in the central part is smaller compared to that in the western part and the mean durations of tremors tend to be shorter, although a larger number of tremors occur here. In the eastern part, the radiated energy varies widely within the area and the number of tremors is quite small. Tremor activities during ETS episodes do not usually propagate across the boundary between the eastern and central parts. However, a few migration sequences crossing the boundary were detected from 2011 to 2014, which might be enhanced by the small L-SSE that bridges the gap between the deeper transition zone and the shallower locked zone^12^.

## Migration pattern of tremor activities, migration speed, and radiated energy

Based on the spatio-temporal evolution of tremor sequences during 28 ETS episodes from 2004 to 2015, the migration pattern of tremor activities can be classified into five categories; (A) a clear eastward migration along-strike in about half of the total ETS episodes (11 episodes)^13^; (B) migration from greater depths in the updip direction, followed by along-strike bilateral migration (10 episodes); (C) a simple westward migration along-strike (2 episodes); (D) complex migration in the strike direction such as eastward migration followed by westward migrations (3 episodes), and; (E) very short migration distance or no apparent migration (the remaining 2 episodes).

**Figure 2:**
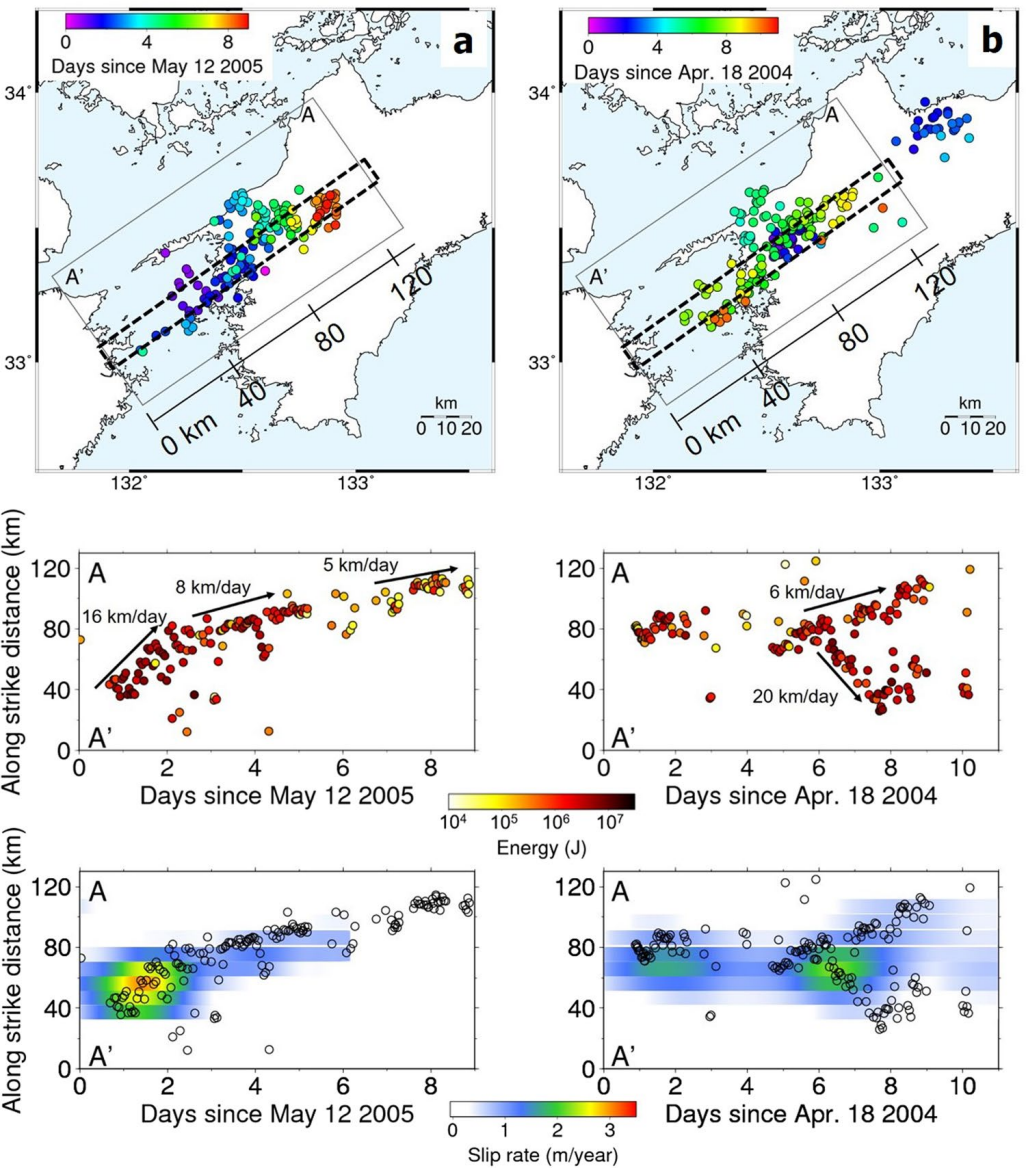
Spatio-temporal evolution of tremor sequences. (a) (upper panel) Map view of tremor activities during the May 2005 ETS episode (category A) with the onset of each tremor sequence shown by color. (middle panel) Tremor activities with radiated energy projected in the strike direction and the estimated migration speed (see Methods). (lower panel) Spatio-temporal evolution of short-term SSEs with a moment magnitude of 6.0 along the cross section enclosed by dashed lines in the upper panel, which is inferred by tiltmeters in ref.^15^. (**b**), Same as (**a**), but in the case of the April 2004 ETS episode in category B. A moment magnitude of short-term SSE during this episode was estimated to be 6.1^15^. Maps are created using the GMT (Generic Mapping Tools, http://gmt.soest.hawaii.edu/) software package^32^.

As a typical example of category A (Fig. 2a), major tremor activity started on May 12 beneath the Bungo channel and then migrated eastward along-strike during the following 9 days. The migration front of the tremors showed a clear parabolic curve, that is, the location of migration front of tremors is proportional to the square root of time. A diffusive process is one plausible mechanism for tremor migration^14^; i.e., the migration speed (see Methods) is greater (~16 km/day) during the initial stage of the ETS episode, and gradually reduces (~5 km/day) as time elapses. The radiated energy of each tremor during a sequence is obviously larger when the migration speed is high, while each tremor radiates a smaller quantity of energy at a lower migration speed.

The migration pattern exhibited by tremor activity during the April 2004 ETS episode is representative of category B (Fig. 2b). The first tremor sequences occurred from April 18 to 20 without clear migration. On April 22, the tremors became active at the downdip side, after which they exhibited an updip migration for 1 day and propagated bilaterally in the strike direction. This westward migration speed is estimated to be ~20 km/day, which is higher than the eastward migration speed of ~6 km/day. The spatio-temporal evolution of the radiated energy (Fig. 2b) indicates that tremors radiate larger amounts of energy during the faster westward migration on average, compared to the slower eastward migration. These observations indicate that the amount of energy released from tremors could be regulated by the speed of the migration front or that faster migration occurs when tremor patches are close to the failure and release greater energy when they fail.

## A positive correlation between migration speed and radiated energy

**Figure 3:**
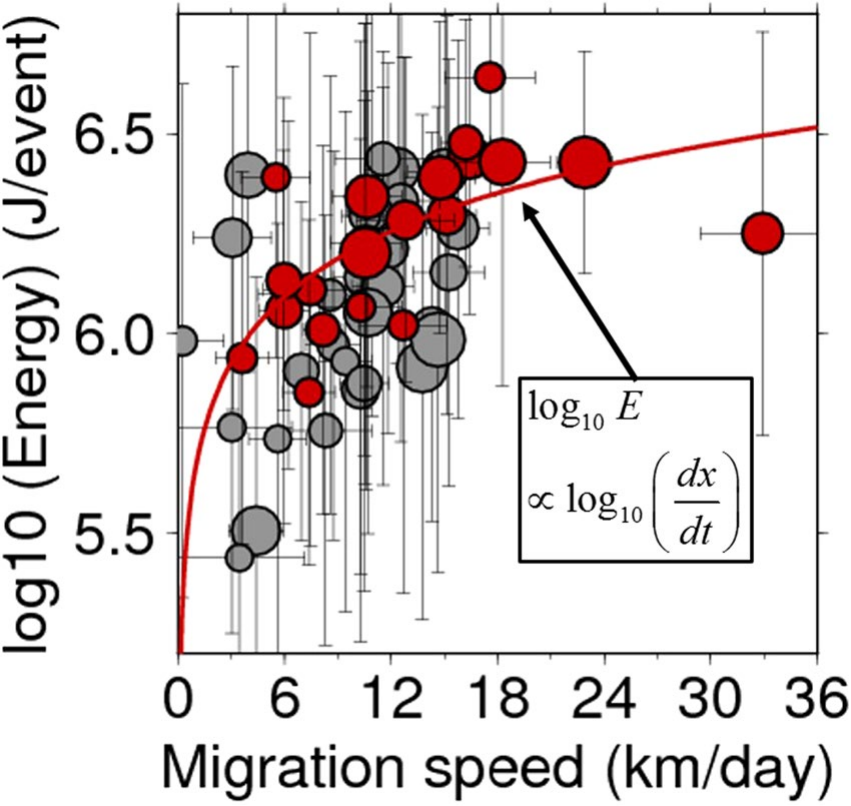
Migration speed and mean energy rate. Migration speed and mean energy in each tremor group including more than 25 events. The magnitude of the symbol shows the number of tremors in each group and the error bar is the 1-σ estimation error for both migration speed and mean energy. Red points correspond to the tremor groups that show a clear parabolic migration, while black points indicate tremor groups whose migration front does not obviously expand in diffusive manner. The red line indicates the analytically obtained relation by fitting all the red points. This figure is created using the GMT (Generic Mapping Tools, http://gmt.soest.hawaii.edu/) software package^32^.

A systematic quantification of migration speed and radiated energy for all 28 ETS episodes (see Methods) revealed that the radiated energy of migrating tremors significantly increases with migration speed (Fig. 3). This positive correlation is less sensitive to the directions of tremor migration. Comparing the radiated energy of tremors with the slip rate on the plate interface obtained by time-dependent finite-fault inversion analyses of two ETS episodes using geodetic measurements^15^, we find that radiated energy of tremors correlate well with the slip rate and that the slip rate becomes faster when the tremor migration speeds increase (Figs 2 and 3). In other words, a plate boundary fault hosting slow earthquakes radiates much more energy as the slip rate of SSE and the migration speed of tremor front during ETS episodes increases.

## Implications for fault model in the Nankai subduction zone beneath western Shikoku

**Figure 4:**
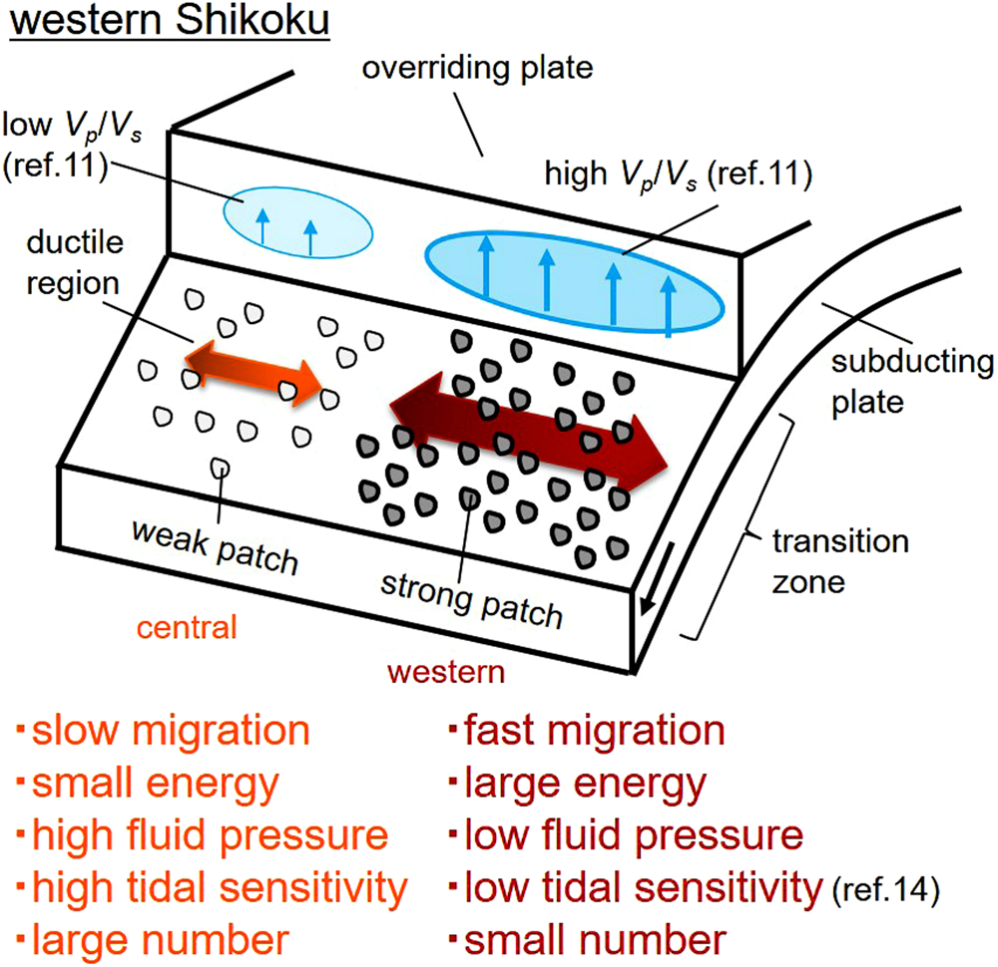
Schematic model of tremor patches at ETS source depth in the Nankai subduction zone. Strong tremor patches caused by low fluid pressure in the western part radiate large amounts of energy, causing fast tremor migration. On the other hand, a high fluid pressure in the central part weakens the strength of tremor patches, resulting in low energy radiation and slow migration.

Based on a theoretical study^9^, tremor migration behaviors are modeled by on-fault stress diffusion regulated by fault slips in the brittle-ductile transition zone. This model assumes a number of brittle tremor patches that lead to unstable slip (slip- or velocity-weakening friction) in the ductile (velocity-strengthening) background fault areas (Fig. 4). This structural heterogeneity generates stress concentration during the stage of fault locking resulting in the driving force of SSEs or tremors. Effective strength is a function of frictional coefficient, normal stress, and fluid pressure, and the effective stress of tremor patches is higher than that of the ductile background region.

In addition, the effective strength in each tremor patch varies with along-strike direction and we refer to the tremor patches with high and low effective strength as “strong” and “weak” patches, respectively. From a geological perspective, brittle lenses such as chert, sandstone, and basalt embedded in mudstones corresponds to the tremor patches in the ductile background^16,17^. Moreover, the effective strength heterogeneity in tremor patches corresponds to the ratio of brittle lenses to the ductile mudstone. The present model uses a combination of brittle and ductile components for generating slow earthquakes, while another models have adopted the transition of frictional behavior from unstable to stable slip above a cutoff velocity^18^ or a conditionally unstable velocity weakening frictional behavior^19^ within the framework of rate and state dependent friction law.

The present model assumes that the effective strength of each tremor patch instantly drops from the peak value to the same (residual) level as the ductile background region. Furthermore, it is assumed that the strength excess, i.e., the increment required to reach the peak strength from initial traction, is uniform in strong and weak patches. This model was originally proposed to describe the observed characteristics that tremors, accompanied by SSEs, initiate energetically in brittle strong patches and migrate in a parabolic form. The along-strike heterogeneity of effective strength in tremor patches is shown to be the essential condition for reproducing the observed parabolic migration of tremors^9^.

If we consider a two-dimensional anti-plane problem in an infinite homogeneous elastic body and the Newtonian rheology, *τ* = *η*(*du*/*dt*), where *τ* is a ductile strength, *η* is a velocity strengthening coefficient, and *du*/*dt* is a slip velocity. The spatio-temporal evolution of shear traction *τ*(*x, t*) from a concentrated source releasing the stress *Δτ* over the length *L* is analytically expressed as

1 $$\tau (x,t)=\eta (du/dt)=\frac{{\Delta }\tau L}{\pi }\frac{(\mu /2\eta ){\rm{t}}}{{x}^{2}+{[(\mu /2\eta ){\rm{t}}]}^{2}}\simeq \frac{\mu {\Delta }\tau L}{2\pi \eta }\frac{t}{{x}^{2}}\equiv D\frac{t}{{x}^{2}}$$

where *μ* denotes the rigidity, *D* is a diffusion coefficient, and the approximation is true if we consider the initial moment of stress diffusion, i.e., *x*^2^>> [(*μ*/2*η*)]^2^ (ref.^9^). Equation (1) gives the following relation for radiated energy $E,E\propto \int {({\partial }^{2}u/\partial {t}^{2})}^{2}dt\propto {x}^{-4}$. The migration front can be defined as the plane *τ* = *τ*_c_, where *τ*_c_ is an average critical strength near tremor front. Using the definition of migration front and Equation (1), the following relation, *τ*_c_*x*^2^ = *Dt*, can be obtained for the migration front. By taking the time derivatives of this relation, the migration speed *dx*/*dt* is found to be proportional to *x*^−1^. Finally, we analytically obtain the positive correlation between energy and migration speed as *E* = *T*(4*τ*_c_^2^/*ηD*)^2^(*dx*/*dt*)^4^, where *T* is a duration of tremor. While this quantitative dependence can be perturbed by the local heterogeneity and the rheology types^9^, this model can qualitatively explain the observed relationship between seismic energy and migration speed, especially for tremor sequences showing a clear parabolic migration (red points in Fig. 3), all of which migrate from the western part to the central part (all 11 ETS episodes in category A in Fig. S2a and the latter half of the December 2011 ETS episode in Fig. S2d). This means that tremor patches in the western part have higher strength, while those in the central part have lower strength (Fig. 4). This along-strike heterogeneity in fault strength explains the fact that tremor front migrating from central to western part or within the central part (black points in Fig. 3) does not obviously expand in diffusive manner (Fig. 2b). Even when we consider all the tremor sequences, the positive correlation between migration speed and radiated energy is found (Fig. 3).

A similar relation has been already documented in the San Andreas Fault zone based on a catalog of low frequency earthquakes (LFEs)^20^. Ref.^20^ focused on the high-speed migration of tremors in the strike direction with a speed of 30–90 km h^−1^ and demonstrated that tremors with high amplitude (~large radiation energy) migrated faster. Considering the analogy between tremor migration in the San Andreas fault and western Shikoku, the stress diffusion model offers one of the plausible mechanisms to explain the positive correlation between the radiated energy and migration speed in tremor episodes.

Figure 4 shows a schematic illustration of a transition zone beneath western Shikoku. A brittle rupture in strong tremor patches in the western part occurs as the initial stage of an ETS episode, radiating more energy (Fig. 1) and migrating in the along-strike direction with a relatively high speed of >10 km d^−1^. Weak patches in the central part then begin to rupture due to the stress transfer with smaller energy and exhibit a slower migration. The contrast of both strong and weak tremor patches in the transition zone results in the diffusive migration of tremors, and the positive correlation between radiated energy and migration speed. Along-strike heterogeneity of fault strength between the western and central part can be validated using the following rupture kinematics with a stress drop *Δτ* migration speed *dx*/*dt*, and average slip rate *V*_*slip*_, such that ${V}_{slip}\,\propto ({\Delta }\tau /\mu )(dx/dt)$^21,22^. This relationship indicates that the ratio of stress drop between the two areas is calculated as the ratio of *V*_*slip*_/(*dx*/*dt*). The average slip rate of the SSE in the western part during the May 2005 ETS episode (Fig. 2a) is estimated to be ~2.2 m yr^−1^ from the slip inversion using tiltmeter records^15^ when tremor front migrates ~16 km/day, while the slip rate of SSE in the central part is ~0.7 m yr^−1^ with a tremor migration speed of ~8 km d^−1^. Substituting these values into the above relationship, the average stress drop in the western part is estimated to be 1.7 times larger than that in the central part. This lateral variation in stress drop agrees well with our conceptual image in western Shikoku (Fig. 4).

Seismic images are highly linked to the strength of tremor patches. Ref.^23^ showed that tremor events accompanied by large SSEs occur where *V*_*p*_/*V*_*s*_ ratio in the overriding plate is high, while those without detectable SSEs are located beneath the low *V*_*p*_/*V*_*s*_ region of the overriding plate, in the Costa Rican subduction zone. The high *V*_*p*_/*V*_*s*_ ratio in the overriding plate implies that fluid from hydrated oceanic crust infiltrates the overriding plate to some extent, and the fluid pressure along the plate boundary consequently reduces, increasing the strength of the tremor patches. On the other hand, the low *V*_*p*_/*V*_*s*_ ratio in the overriding plate indicates abundant fluids are possibly trapped within the plate boundary due to a low permeability barrier, resulting in weak tremor patches.

Similar significant along-strike difference of *V*_*p*_/*V*_*s*_ ratio in the overriding plate was inferred between the western and central parts in western Shikoku^11^. The bottom of the overriding plate (3 km above the subducting Philippine Sea plate) in the western part is characterized by a relatively high *V*_*p*_/*V*_*s*_ ratio, which indicates that leakage of fluids into the overriding plate results in a decrease in fluid pressure, generating higher strength patches in the western part, compared with the central part.

When plenty of fluid exists, tremor patches can be easily ruptured by external stress perturbations due to the low critical stress level. In fact, tremors in the central part have been shown to be more sensitive to the tidal effect than those in the western part^14^. Such a high sensitivity of tremor patches to external stress perturbations leads to a high rate of tremor production in the central part (Fig. 1b), suggesting a negative correlation between the migration speed and the number of tremors. A similar negative correlation is also observed in the 2010 ETS episode along the Cascadia subduction zone^24^. Therefore, the model of the along-strike heterogeneity in the strength of tremor patches in western Shikoku provides a unified explanation for the lateral variation in the tremor density (Fig. 1b), radiated energy (Fig. 1a) and its relation to migration speed (Fig. 3) as well as the previously obtained features of the fluid distribution and the tidal sensitivity of tremors.

Fluid migration is an alternative model that explains the positive correlation between radiated energy of tremors and their migration speed, if the strength of the patch is uniform (Fig. 3). Namely, faster migration of fluid triggers the brittle failure of a larger number of tremor patches resulting in the radiation of more energy. This model of heterogeneous fluid migration speed qualitatively explains the positive correlation without a heterogeneity in fault strength. However, this model also implies that the fluid migration speed positively correlates with the number of tremors, which is different from the observed feature seen in the tremor density map in western Shikoku (Fig. 1b). In addition, the fluid migration model requires that pressurized fluids be presumably trapped on the fault where tremor migration initiates, and when tremors occur in this area, the fluids start to diffuse in the surrounding areas as the tremor migrates. This model expects higher fluid pressure in the area of tremor initiation, and consequently results in smaller slip and lower stress drop therein, which contradicts the fact that tremors radiate more energy and the fault slips faster in the initial stage of each ETS episode (Fig. 2). Therefore, fluid migration alone is not sufficient but stress diffusion mechanism (or hybrid model for both stress and fluid migration) is necessary to explain all the features of tremor migration and the relation between radiated energy and migration speed, as observed in the present study.

In terms of the along-strike heterogeneity in radiated energy of tremors, the present study suggests that the strengths of tremor patches on the subducting plate are controlled by the physical properties related to fluid existence in the overriding plate as indicated from the seismic structure^11^. The comprehensive exploration of tremor activities, including radiated energy, will enable us to globally map the spatial distribution of the strength of tremor patches in subduction zones where slow earthquakes occur. Physics-based numerical simulations considering such a realistic along-strike heterogeneous distribution of tremor strength in transition zones would contribute to a better quantitative understanding of the mechanisms of slow earthquakes.

## Methods

The present study estimates migration speed and mean energy rate of tremor activities during ETS episodes in western Shikoku based on a centroid tremor catalog derived by ref.^10^, which can be downloaded from “Slow Earthquake Database” (http://www-solid.eps.s.u-tokyo.ac.jp/~sloweq/)^25^. This tremor catalog is constructed using the procedure described below for understanding the tremor activities in terms of radiated energy. It consists of the hypocenter locations and radiated energy estimated based on the newly developed algorithm and the borehole data of the High Sensitivity Seismograph Network (Hi-net)^26^ operated by the National Research Institute for Earth Science and Disaster Resilience (NIED). First, tremor sequences are extracted by cross correlation analysis based on the root-mean-square (RMS) envelope waveforms. The extracted signals can be used to determine source locations based on the envelope correlation method^1^ so that a source location is individually determined every two minutes. The source locations estimated during a period of successive one hour are clustered into one or two centroid tremor locations using the *k*-means approach^27^. The radiated energy is estimated for each sequence based on the observed amplitude using the procedure of ref.^7^, independent of the source estimation. This algorithm enables the evaluation of the radiated energy of tremors more accurately than the previously adopted algorithm^28^, which determines the source locations of tremors based on both observed energy and differential travel times among stations. The previous algorithm sometimes suffers from determining accurate source locations of tremors with high amplitude because of their complex waveforms originating from a finite source area. Thus, it results in the underestimation of energy. Since this algorithm extracts the tremor sequences before determining source locations, it can reduce instances in which tremor signals are missed. In fact, the total energy in western Shikoku based on the new catalog is two to three orders larger compared to that obtained by the previous one^28^.

The catalog includes a total of 28 ETS episodes (4,376 tremor counts) from April 2004 to March 2015. The recurrence interval of ETS episodes is ~six months^6^ with some fluctuations of adjacent long-term SSEs (L-SSEs) in the Bungo channel^29^. During each ETS episode, along-strike and/or dip migration of tremors are almost always observed^30^. Based on the spatio-temporal evolution of the tremor front, the migration speed is objectively estimated as follows. First of all, we define initial tremors (Fig. S1a). A tremor that is first detected in each bin in the along-strike (dip) direction with 10 km length (width) is a candidate for an initial tremor. If more than five tremors are detected within one day from the occurrence of the candidate, the candidate is defined as the initial tremor in the bin. Otherwise, the secondary detected tremor is taken as the next candidate, and then we evaluate to determine whether the candidate is the initial tremor or not. This procedure is repeated until the initial tremor in each bin is defined. If all tremors in the bin do not satisfy the condition, we ignore these tremors. Once the initial tremors are determined, tremors within one and a half days following the initial tremor in each bin are categorized as a tremor front (Fig. S1b). Tremor fronts are separated into groups with an interval of two days from the first detection of the tremor front (Fig. S1c). Here, we ignore groups containing less than 10 tremors. Finally, migration speed is estimated as the slope by fitting the linear function to each group using the least square approach (Fig. S1d). Mean energy in each group is also estimated by averaging the energy of all tremors in the common logarithmic scale. An example extraction of the migration front is summarized in Fig. S1.

## Electronic supplementary material

Figures S1 and S2
