# Supplementary material for: Strength of tremor patches along deep transition zone of a megathrust
Source: Sci Rep. 2018 Feb 26;8:3655. doi: 10.1038/s41598-018-22048-8 (PMC5826920; doi:10.1038/s41598-018-22048-8)
Supplement: Supplementary file 1 — Figures S1 and S2 [file 41598_2018_22048_MOESM1_ESM.docx]

Strength of tremor patches along deep transition zone of a megathrust

Masayuki Kano^1,2*^, Aitaro Kato^1^, Ryosuke Ando^3^ and Kazushige Obara^1^

^1^Earthquake Research Institute, The University of Tokyo, Tokyo, Japan.

^2^Now at Department of Geophysics, Graduate School of Science, Tohoku University, Sendai, Japan.

^3^Department of Earth and Planetary Science, The University of Tokyo, Tokyo, Japan.

*Corresponding author: Masayuki Kano (email: kano@zisin.gp.tohoku.ac.jp)

Supplemental information

**
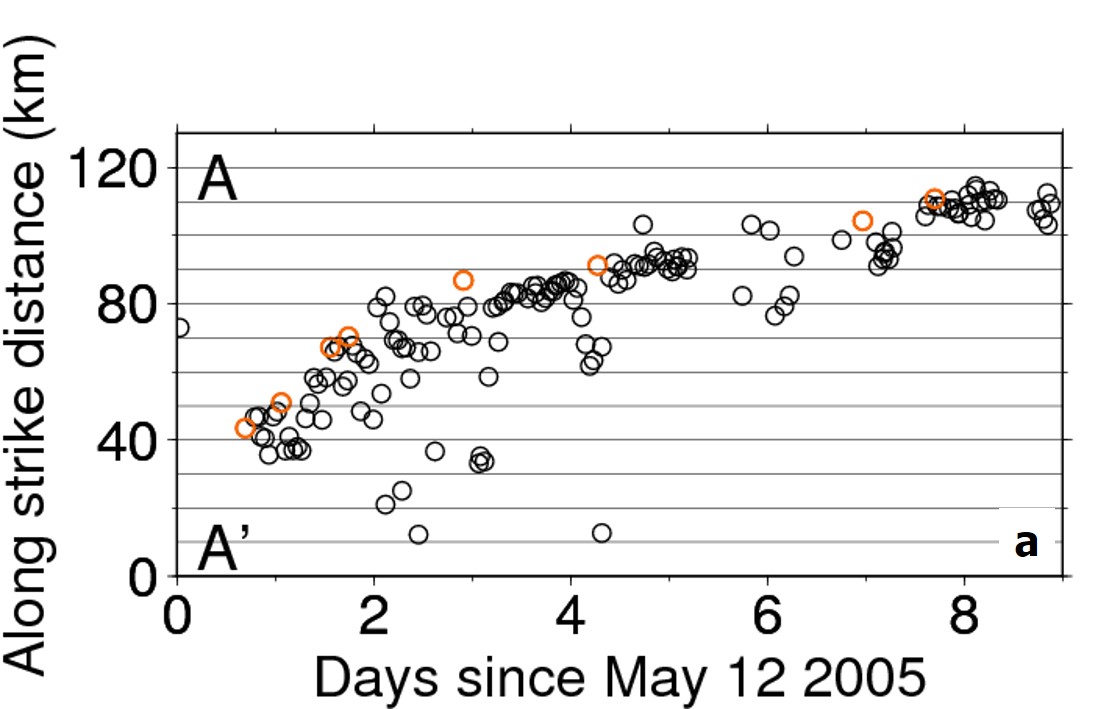

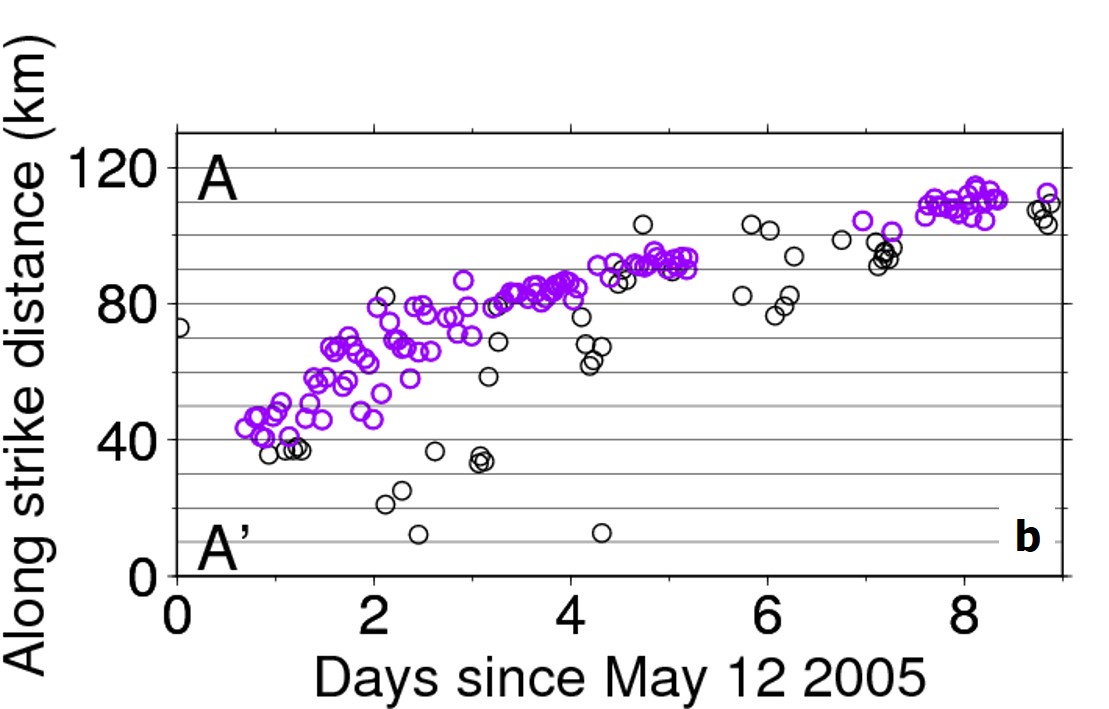

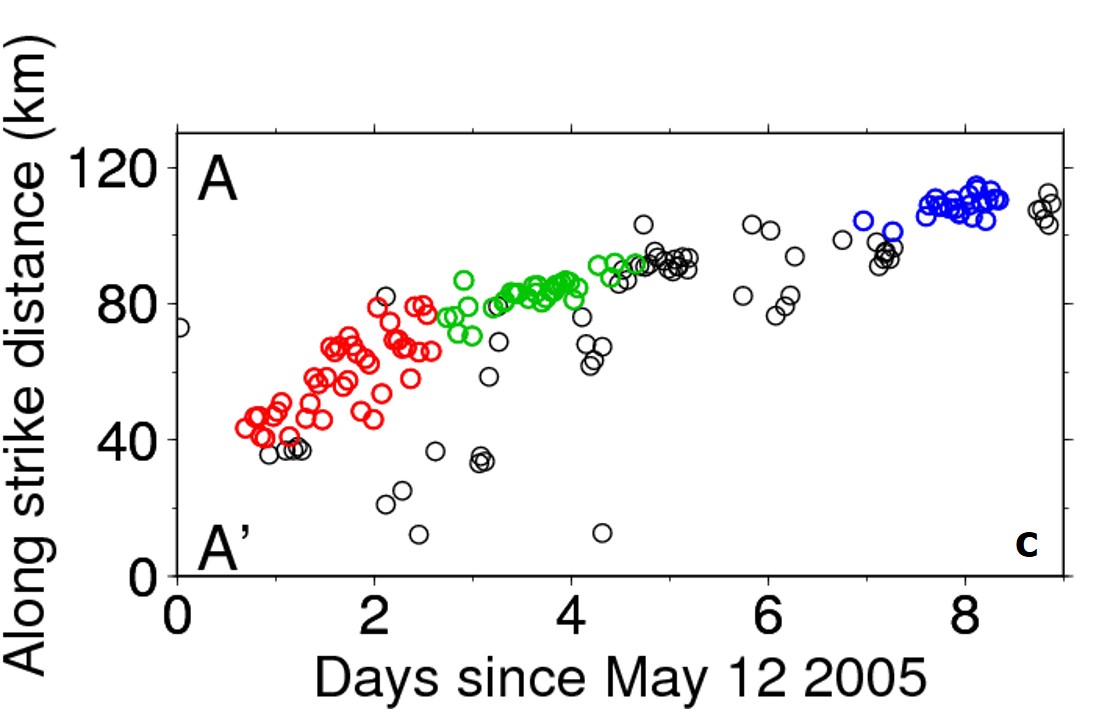

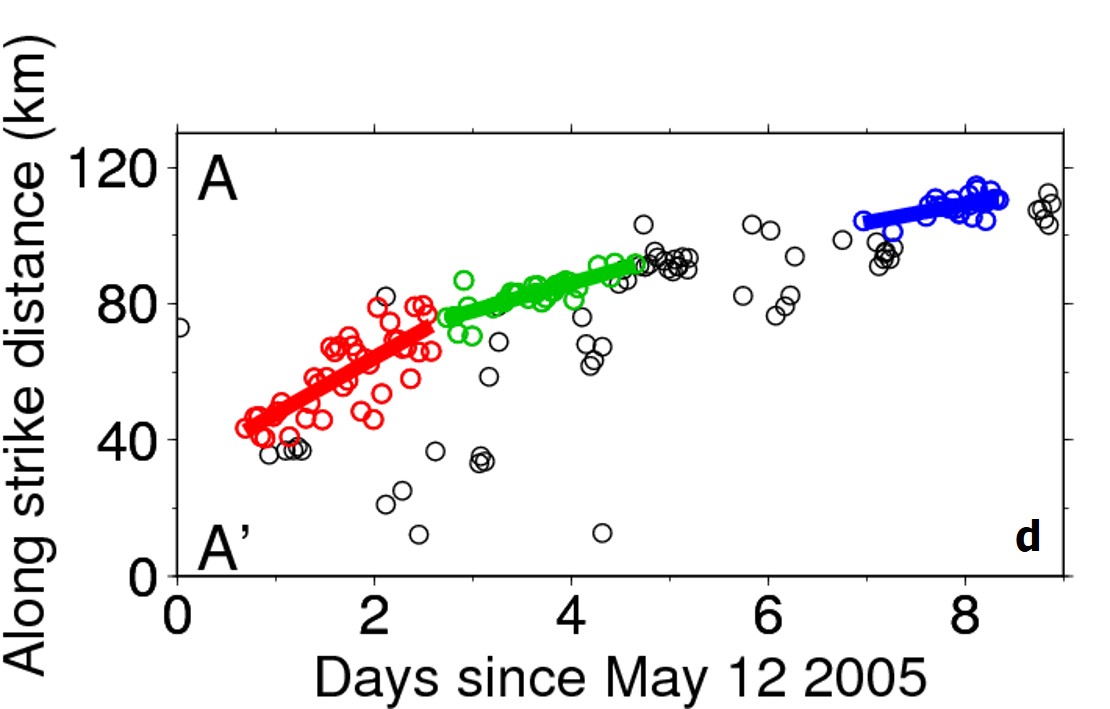
**

**Figure S1.** Estimation of migration speed and mean energy rate in the case of the May 2005 ETS episode. Black circles indicate tremor locations estimated by ref. 10. First, we determine the initial tremors in each bin in the along-strike direction with 10 km length colored by orange in **a**. The tremor front is defined as tremors that occurred within 1.5 days from the initial tremor in each bin shown by purple in **b**. Tremors in the tremor front are divided into three groups (red, green, and blue in **c**.) with a time interval of two days from the first detection of tremor front. Finally, we estimate the migration speed as the slope by fitting the linear function to each group based on the least square approach. The mean energy is calculated by averaging the radiated energy of all tremors in each group in the common logarithmic scale.

**a.**


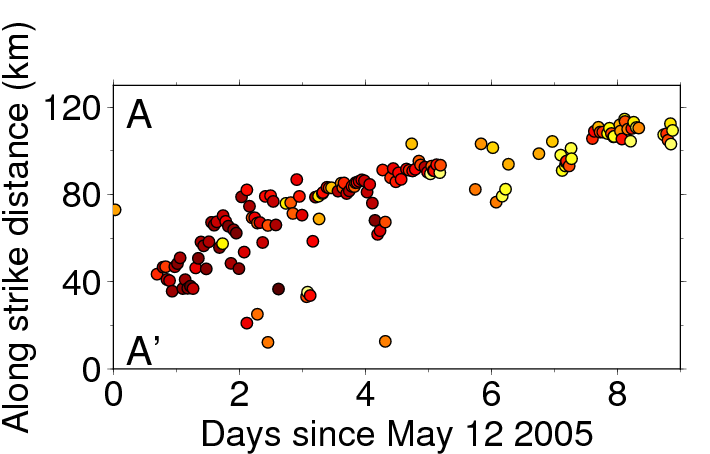

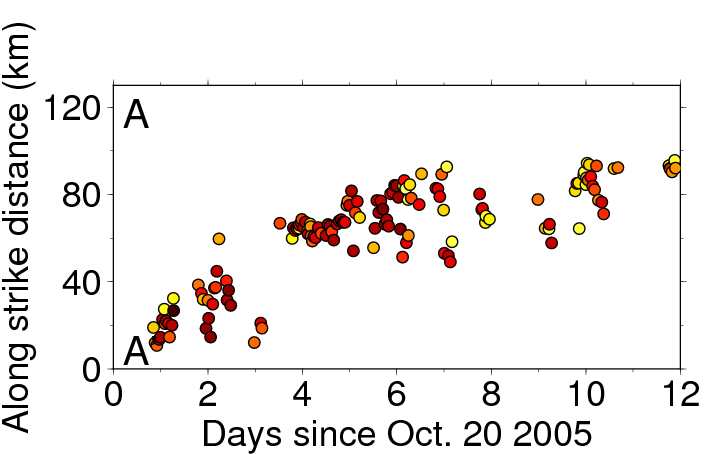

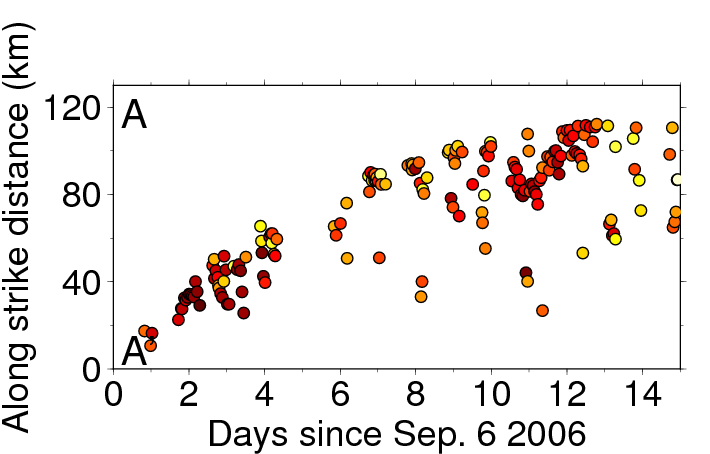

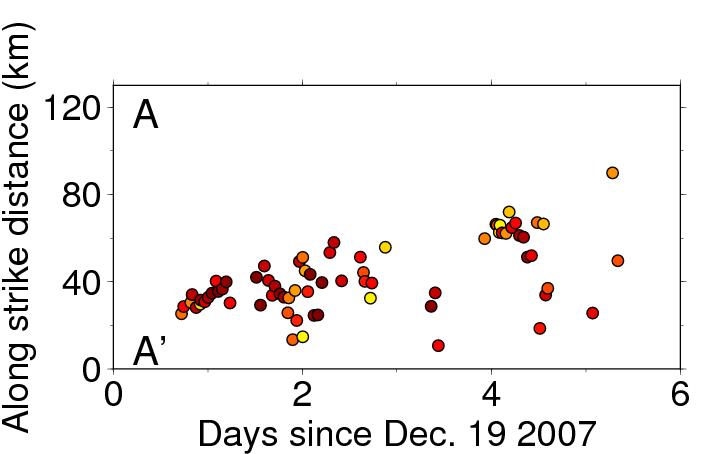

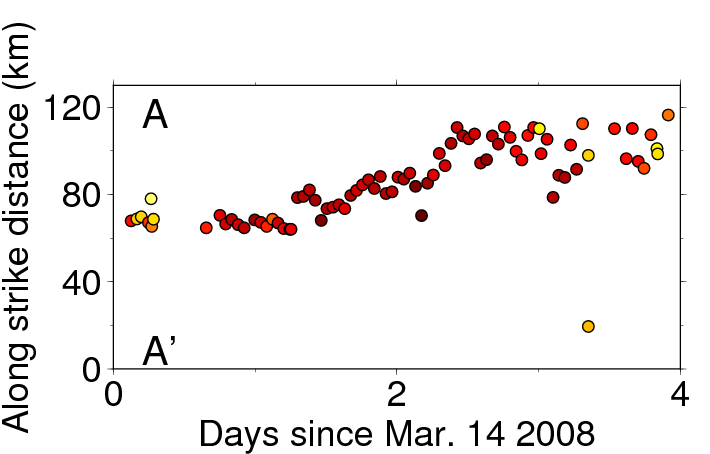

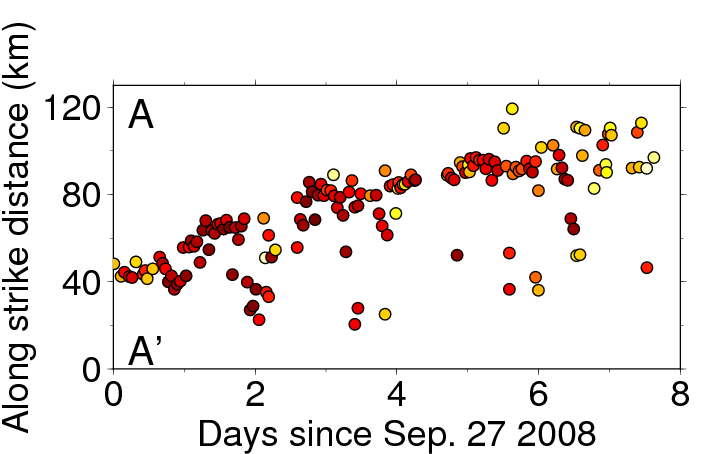

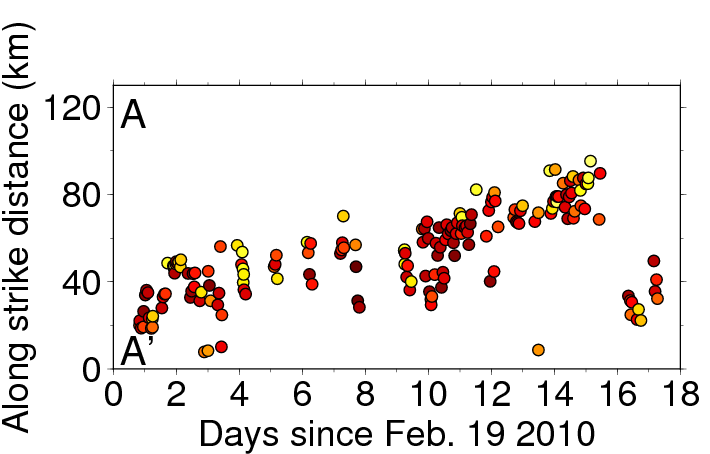

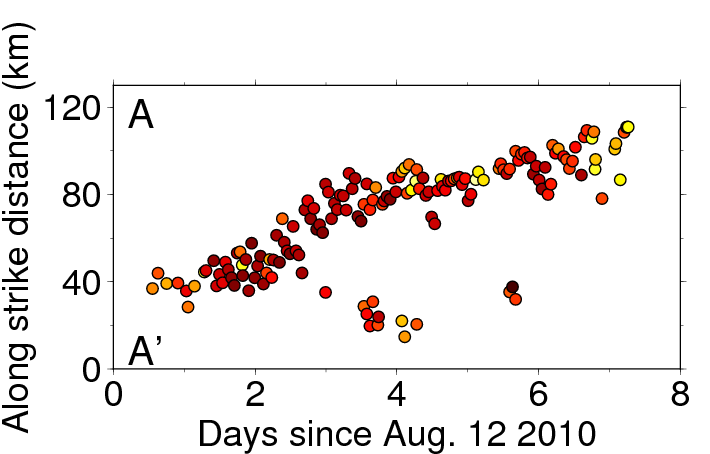

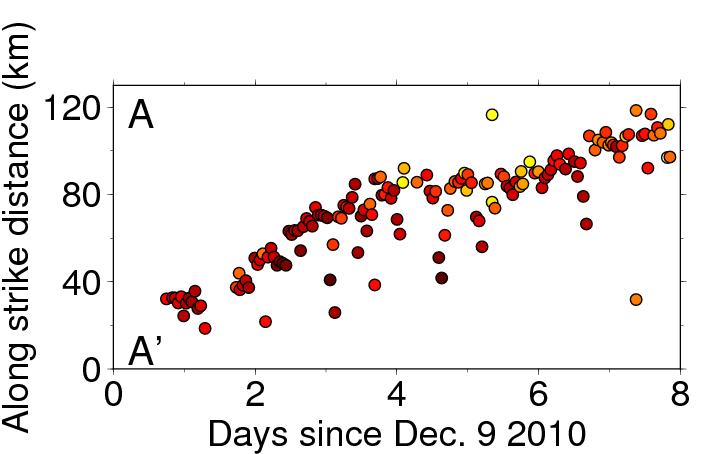

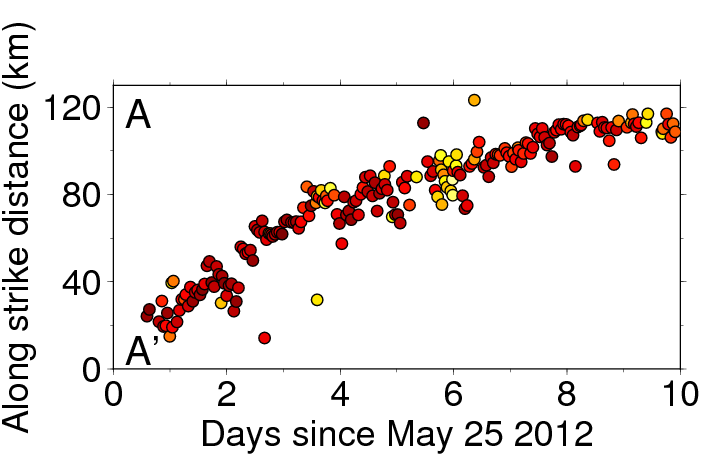

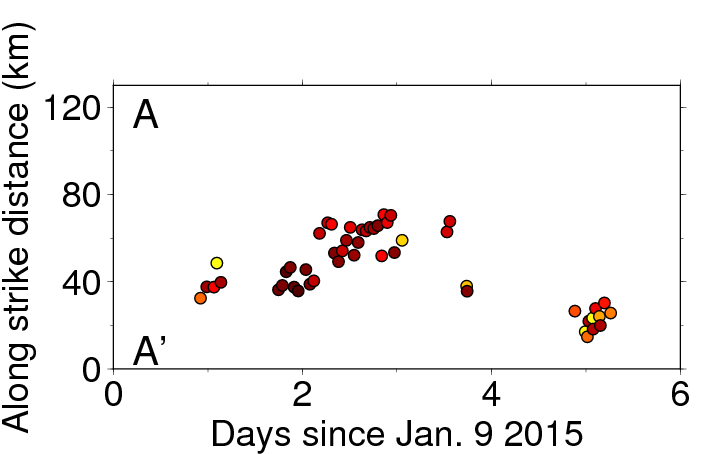


**b.**


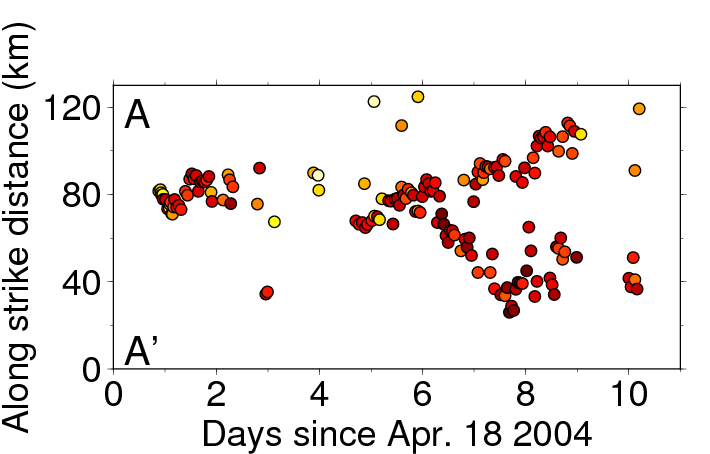

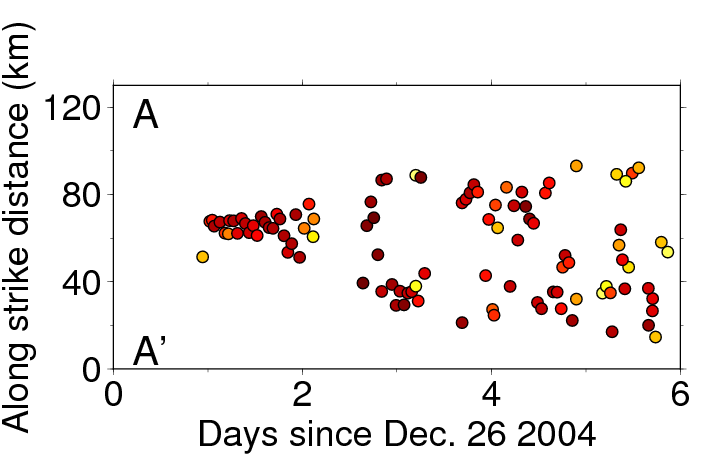

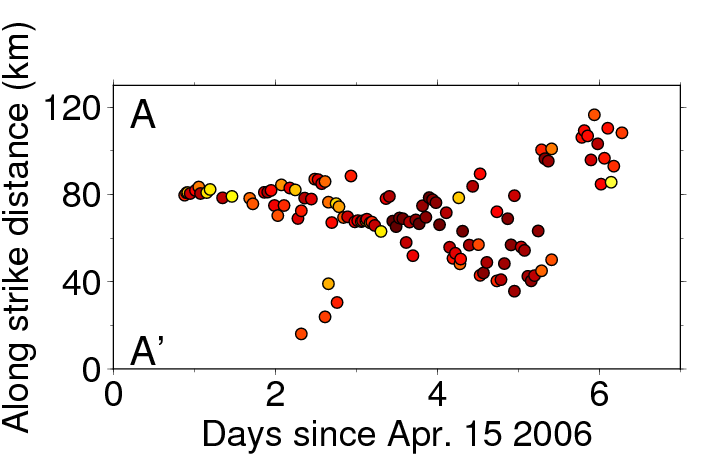

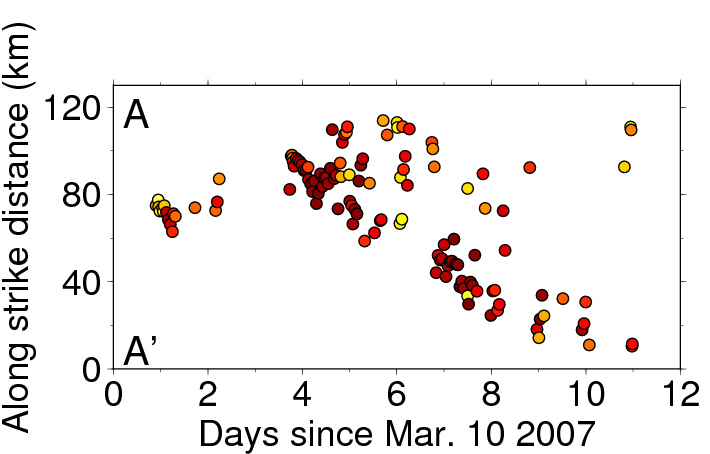

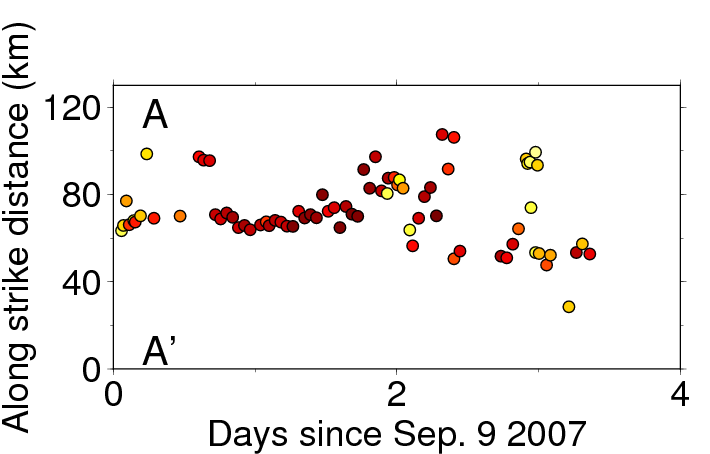

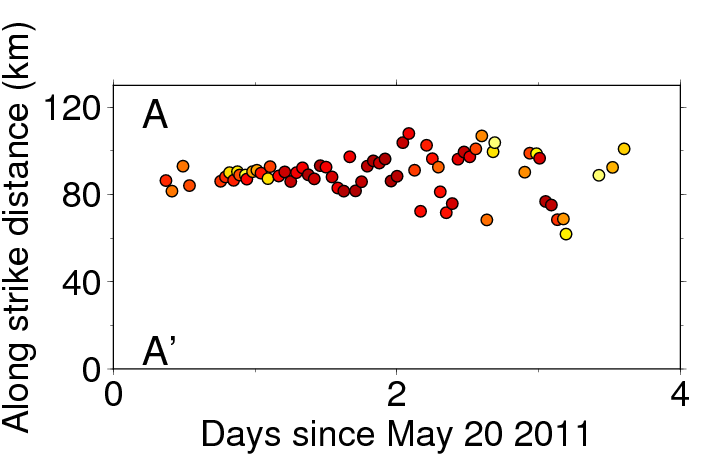

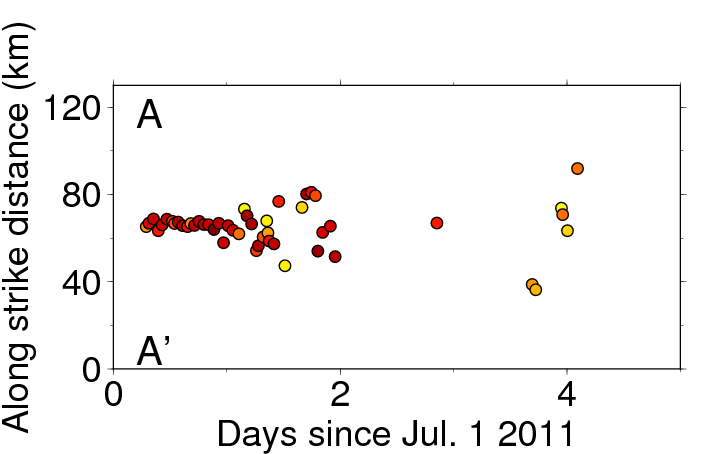

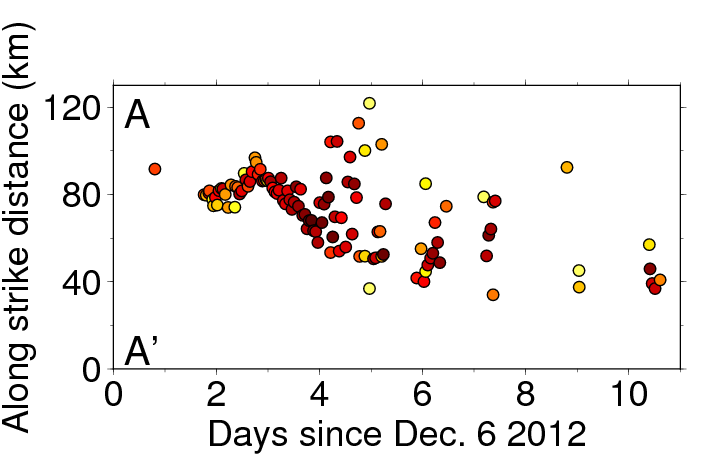

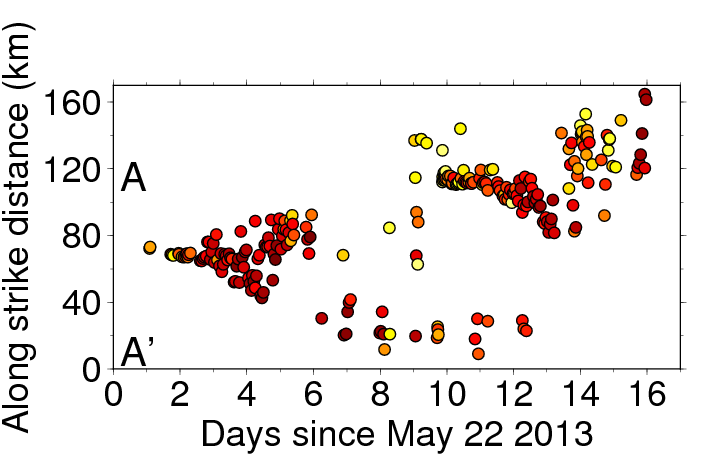

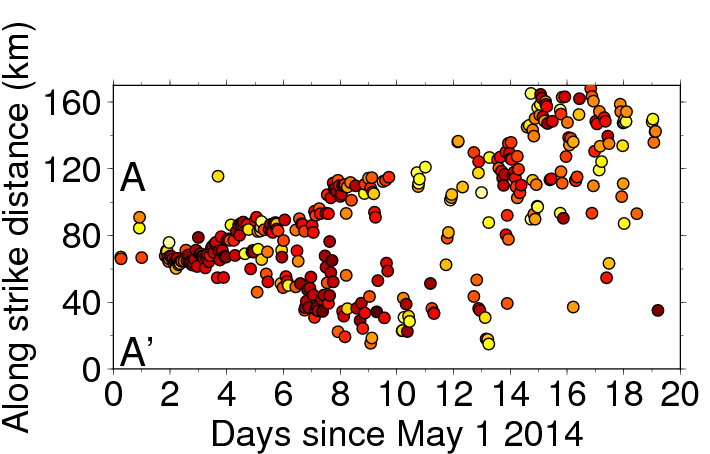


**c.**


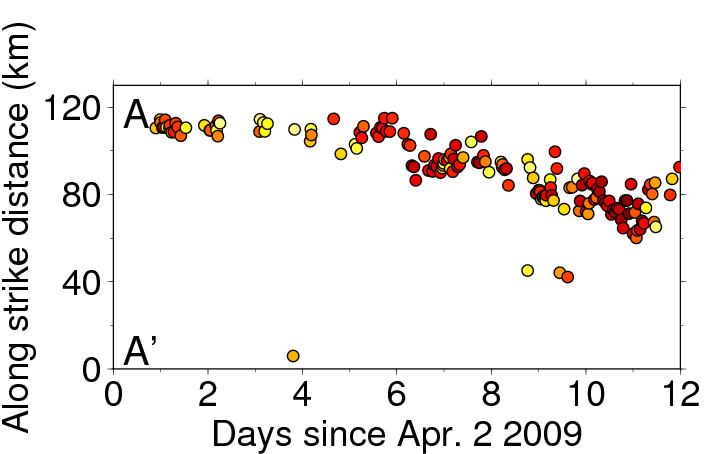

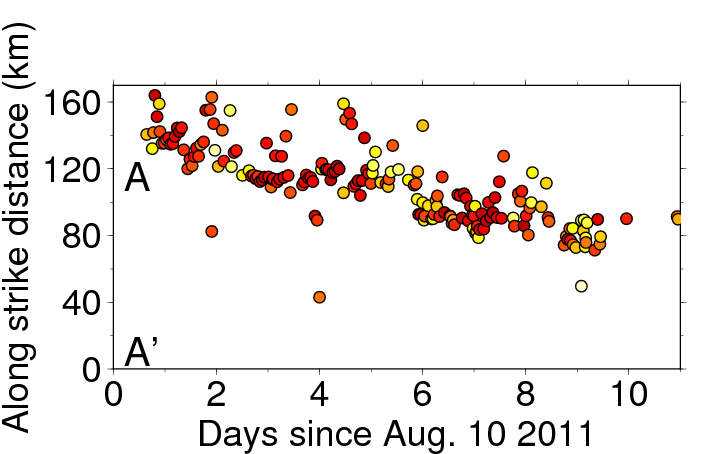


**d.**


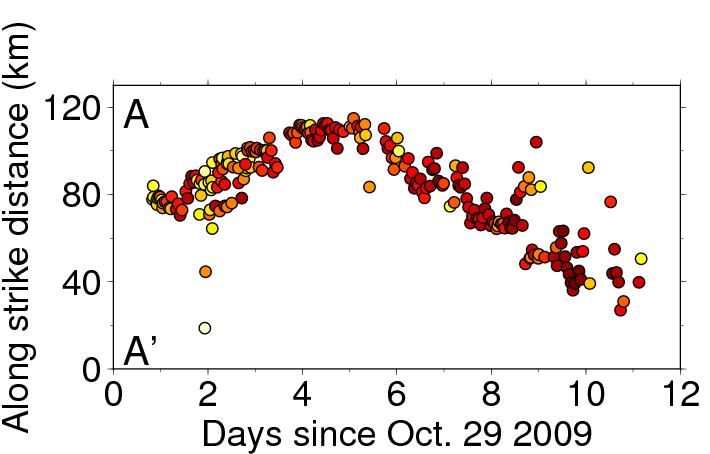

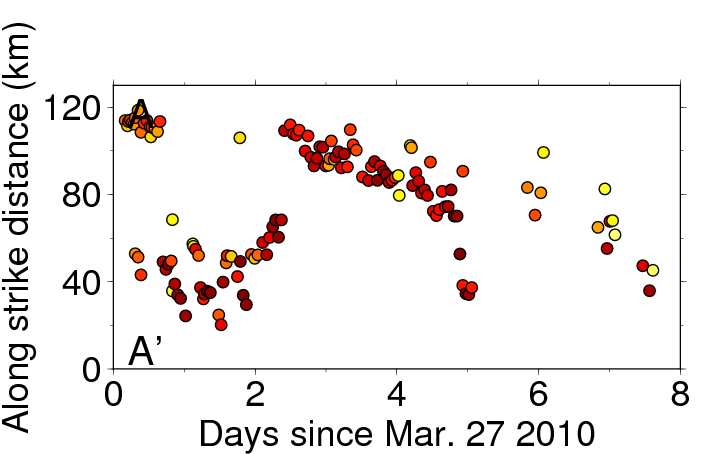

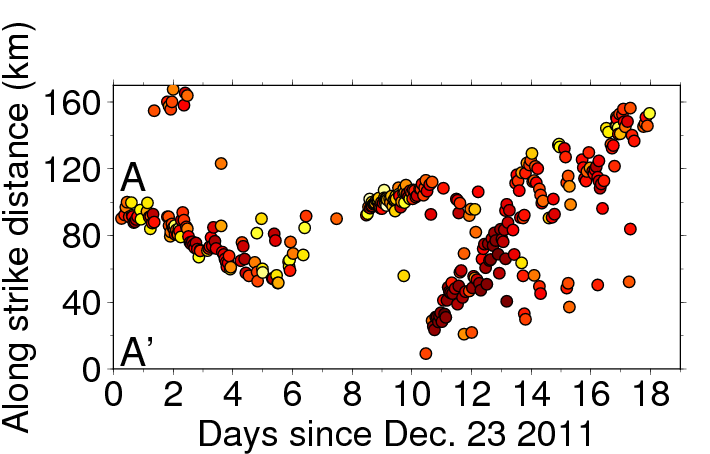


**e.**


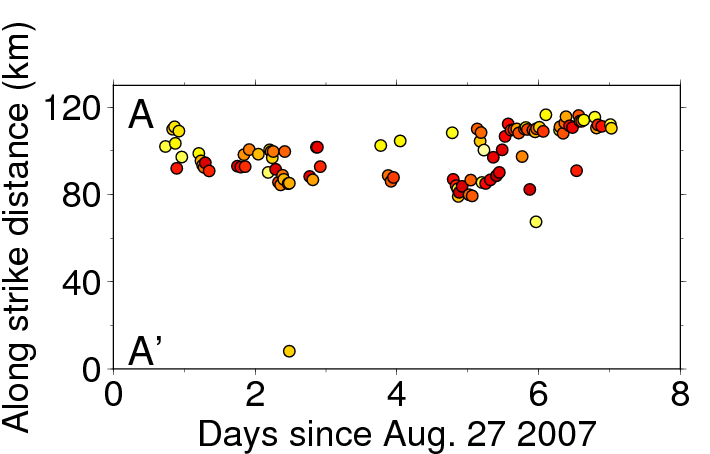

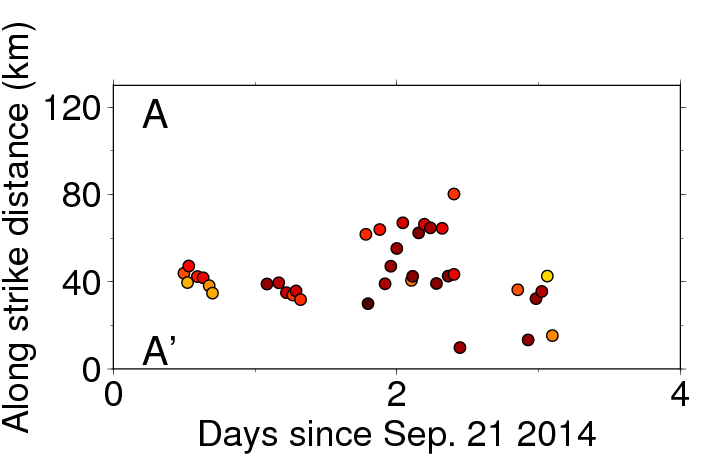

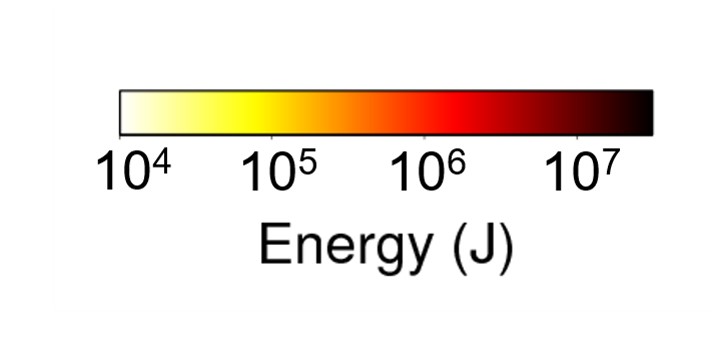


**Figure S2.** Spatio-temporal evolution of tremor sequences with radiated energy projected in the along-strike direction shown in Fig. 2 during the ETS episodes from 2004 to 2015, each of which is categorized in **a,** (A) – **e,** (E) in the main text.
